# CAPRICHO: Interpretable Quality Flagging and Flexible ChEMBL Bioactivity Curation for QSAR Modeling

**DOI:** 10.1021/acs.jcim.6c00802

**Published:** 2026-09-23

**Authors:** David Alencar Araripe, Srijit Seal, Olivier J. M. Béquignon, Gerard J. P. van Westen

**Affiliations:** † Division of Medicinal Chemistry, Leiden Academic Centre for Drug Research, Leiden University, Einsteinweg 55, 2333 CC Leiden, The Netherlands; ‡ Department of Human Genetics, Leiden University Medical Center, Einthovenweg 20, 2333 ZC, Leiden, The Netherlands; § Human Chemical Company, San Francisco, California 94129, United States; ∥ Department of Pharmaceutical Biosciences and Science for Life Laboratory, Uppsala University, Uppsala 75237, Sweden; ⊥ Broad Institute of MIT and Harvard, Cambridge, Massachusetts 02142, United States; # Centre for Molecular Informatics, Yusuf Hamied Department of Chemistry, 2152 University of Cambridge, Cambridge CB2 1EW, U.K.; ∇ STAR-UBB Institute, Babeş-Bolyai University, Cluj-Napoca 400084, Romania

## Abstract

Preparing ChEMBL bioactivity data for quantitative structure–activity relationship (QSAR) modeling requires navigating curation decisions that often lack documentation and are hard to reproduce. We present CAPRICHO (ChEMBL Aggregation Package with Robust Inspection and Curation Handling Options), a Python command-line tool addressing two gaps in current workflows: transparency and flexibility. Unlike approaches that remove problematic data, CAPRICHO flags quality issues while preserving all data for inspection, letting users assess how each curation decision affects cross-assay comparability. Aggregation is configured via customizable grouping columns, supporting both traditional QSAR and emerging assay-aware modeling paradigms, with all parameters recorded in recipe files for reproducibility. Three case studies demonstrate the tool: (i) programmatic support of ChEMBL max-curation standards, (ii) CYP inhibition multitask curation, and (iii) Caco-2 permeability with unit standardization. Together, these show that preserving rather than removing flagged data lets practitioners identify which quality issues most affect comparability and balance data quality with availability for QSAR modeling. CAPRICHO is freely available at https://github.com/CDDLeiden/Capricho

## Graphical abstract

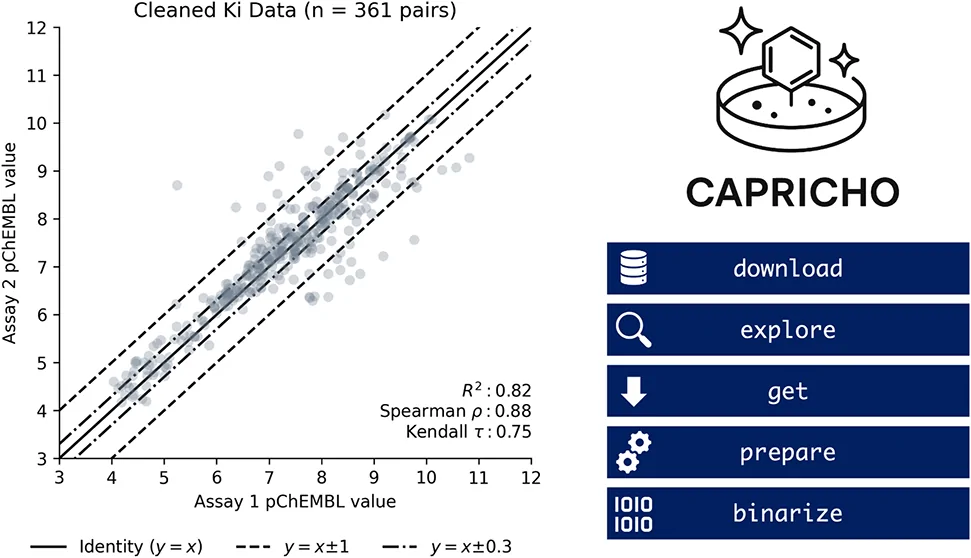

## Introduction

Public bioactivity databases enable a widespread usage of quantitative structure–activity relationship (QSAR) models for compound property prediction.^1,2^ ChEMBL has become an invaluable resource for training such models, providing carefully curated compound-target measurements.^3–5^ Yet, the definition of appropriate curation standards varies across modelers depending on project requirements and data availability, and curated QSAR data sets published in the literature often lag behind the latest ChEMBL releases. Recent efforts have improved reproducibility through standardized SQL queries and benchmarking protocols tied to specific ChEMBL releases,^6^ but they still require further compound standardization, such as removal of salts or organic solvents and identification of tautomers.^7^

Training QSAR models involves choices such as which activity types to include, how to handle similar but distinct assay types, and how to resolve conflicting readouts. While commonplace in the field, novice modelers may lack expertise to navigate such decisions and their potential pitfalls. Landrum and Riniker,^8^ showed that combining data across assays demands careful quality control, with IC_50_ showing higher variability than K_i_, consistent with prior studies on ChEMBL data showing that IC_50_ measurements are inherently assay-condition-dependent.^9,10^ Separately, Tetko et al.^11^ showed that compounds registered both with and without defined stereochemistry produced duplicates during aggregation, artificially inflating evaluation metrics. Despite various curation workflows addressing these issues,^8^ no single tool has consolidated these approaches for broader usage.

An underappreciated issue in QSAR data curation is the lack of transparency on removal decisions. Most existing workflows silently drop data that fails curation criteria, leaving no record of what was removed or why. Practitioners therefore cannot learn from curation trade-offs or identify cases where standard criteria may not apply. Existing aggregation pipelines also lack the flexibility to experiment with modeling techniques that incorporate assay metadata as input features, as performed by Schoenmaker et al.^12^

We developed CAPRICHO (ChEMBL Aggregation Package with Robust Inspection and Curation Handling Options), a Python package guided by two principles: transparency and flexibility. Inspired by the Portuguese word *capricho* (doing something meticulously, with care and attention to detail), the package flags problematic data rather than dropping it, preserving both filtered and retained entries for inspection. Each quality flag is therefore interpretable, letting users visualize and quantify its effect on cross-assay comparability before removal. Curation decisions are exposed as parameters and recorded in configuration files for independent verification. Aggregation flexibly supports additional metadata (e.g., ChEMBL assay descriptions, cell types) as grouping variables, both improving cross-assay comparability and facilitating models that use this metadata as input features.

## Methods

CAPRICHO is available as a Typer-based (https://typer.tiangolo.com/) command-line tool with shell completion. Five commands make up the interface: download (fetch the ChEMBL local backend, or register a ChEMBL SQLite release already present on the system), explore (interactive exploration of the database, including direct SQL queries and schema inspection), get (main data curation and aggregation pipeline), prepare (filtering bioactivity points based on quality criteria), and binarize (convert activities to binary labels for classification tasks).

### Workflow

The get routine runs in two stages: retrieval and processing, then flexible aggregation. Two backends share identical processing logic: a local SQL database (via chembl_downloader)^6^ for fast queries, or the ChEMBL web API (via chembl_webresource_client) requiring no local storage at the cost of slower retrieval.

### Data Processing and Quality Control

After retrieval, SMILES are standardized via chembl_structure_pipeline,^14^ with an additional regex step that removes, per SMILES component, exact matches to a documented list of salts and solvents not stripped by the structure pipeline (Supporting Text S5). Original ChEMBL SMILES are kept for reference (canonical_smiles). Problematic entries are flagged in four categories: structural (missing SMILES, mixtures after salt removal, undefined stereochemistry), assay (size limits, insufficient overlap), measurement (incompatible units for conversion [e.g., %, μg mL^–1^, cm/s, and hour when converting Caco-2 activity readouts to CAPRICHO’s default 10^–6^ cm/s for permeability units], unit annotation errors [activity pair on the same molecule and target with a pChEMBL differing by an exact multiple of 3.0 log units]), and source metadata (missing dates).

CAPRICHO also flags apparent conflicts between annotations: when activity_comments indicate inactivity or an inconclusive result but standard_relation is “=”, the source relation and value are preserved, and the combination is flagged for user review. CAPRICHO also performs cross-document duplicate detection by identifying identical compound-activity pairs (same molecule, same pChEMBL value) appearing in multiple documents, a pattern often arising from data citation that may not be detected by ChEMBL’s native potential_duplicate annotation. While not directly affecting target affinity, it can inflate cross-assay concordance or apparent confidence in a measurement.

All entries are preserved in the output. Processing steps applied by CAPRICHO (salt removal, standard_relation correction, cross-document duplication, pChEMBL calculation) are tracked in a data_processing_comment column, whereas quality flags remain registered in data_quality_comment. If applicable, multiple comments are appended with “&” and are never overwritten. Users can inspect why specific entries were excluded and choose removal criteria suiting their modeling goals. Lastly, get and prepare commands report absolute counts and percentages for the affected measurements and retained aggregated data points.

### Flexible Aggregation

Compound equivalence supports five methods via --compound-equality: SMILES (stereosensitive), connectivity (first 14 characters of the InChIKey; stereoinsensitive), full InChI, full 27-character InChIKey, or fingerprint (stereosensitive). The fingerprint method concatenates Morgan (radius = 2) and RDKit (minPath = 1, maxPath = 7) fingerprints which encode complementary information (local atom environments versus linear substructure paths). Fingerprint-based equivalence ignores stereochemistry by default and can encode it via --chirality argument, since the Morgan fingerprints support stereosensitivity (unlike the RDKit path fingerprint).

Aggregation accepts arbitrary metadata columns as grouping variables through --id-columns. By default, data aggregates across all assays for the same compound-target pair. Users can enforce finer separation by adding metadata fields (e.g., including assay_type prevents combining binding and functional data). Protein variants are treated as distinct entities by default since mutations can significantly affect assay readouts. This can be disabled via --aggregate-mutants. The flexibility supports traditional QSAR approaches, assay-aware modeling strategies,^12^ and multitask settings where activity types (e.g., IC_50_, K_i_) become separate tasks to leverage data availability while maintaining their distinct measurement characteristics. Rows kept separate but sharing the selected compound identifier and target are labeled with shared_identifier_group for downstream inspection whenever applicable (e.g., when activity points measured on mutants yield a “duplicated” value – one compound affinity reported on a mutation and on a wild type target).

Repeated measurements for the same compound-target pair undergo statistical aggregation. For censored measurements (standard_relation ≠ “=”), aggregation occurs only when both the relation and pChEMBL value match exactly, preventing inappropriate combinations of different upper or lower bounds from independent assays. Output follows a pipe-separated format similar to Papyrus,^7^ preserving all original values (pChEMBL, SMILES, identifiers, relations) alongside computed statistics. Geometric statistics (mean, median, SD, MAD, counts) are computed for pChEMBL, while arithmetic statistics are used for standard_value aggregation. This enables measurement-level filtering after aggregation: prepare can remove individual flagged measurements from pipe-separated columns and recompute statistics without discarding the whole compound-target row.

### Available Parameters

Table 1 summarizes the key parameters available in CAPRICHO’s get command, organized by functional category.

**Table 1:** Key Parameters for CAPRICHO’s Data Retrieval and Aggregation Pipeline (capricho get)

category	parameter	description	default
data selection	--target-ids	ChEMBL target IDs	—
	--assay-ids	ChEMBL assay IDs	—
	--molecule-ids	ChEMBL molecule IDs	—
	--confidence-scores	confidence scores (1–9)	7,8,9
	--bioactivity-type	activity types (e.g., K_i_, IC_50_, EC_50_)	all
	--assay-types	assay types: B(inding), F(unctional), A(DMET), etc.	B,F
	--standard-relation	filter by relation (=, < , > )	=
	--standard-units	filter by measurement units	—
compound identity	--compound-equality	method: smiles, inchi, inchikey, connectivity, or mixed_fp (fingerprints)	connectivity
	--chirality	consider stereochemistry in fingerprints	false
	--drop-unassigned-chiral	flag compounds with undefined tetrahedral stereocenters	false
aggregation control	--id-columns	additional columns appended to the aggregation key (e.g., standard_type)	—
	--value-column	column holding the measured value to summarize: pchembl_value or standard_value	pchembl_value
	--aggregate mutants	pool wild-type and mutant data	false
quality filters	--min-assay-size	minimum compounds per assay	—
	--max-assay-size	maximum compounds per assay	—
			
	--require-doc-date	exclude entries without document dates	false
	--strict mutant-removal	flag assays with mutation keywords	false
analysis filters	--min-assay-overlap	minimum compounds shared between assays (for reproducibility of the analysis by Landrum and Riniker^8^)	0
data processing	--calculate-pchembl	calculate pChEMBL if not reported	false
	--convert-units	standardize units (permeability, concentration, etc.)	false
	--curate-annotation-errors	correct standard_relation for censored comments	true
backend and output	--chembl-backend	downloader (local SQL) or webresource (API)	downloader
	--chembl-version	specific ChEMBL version	latest
	--skip-not-aggregated	skip saving preaggregated data file	false
	--skip-recipe	skip saving JSON recipe file	false

## Results and Discussion

### Case Study 1: Transparent Quality Control for Cross-Assay Aggregation

A key challenge in curating ChEMBL data for QSAR modeling is that the data is often derived from assays with a varying number of research protocols, institutes, and executed by different researchers. Landrum and Riniker demonstrated that a naive combination of K_i_ or IC_50_ measurements introduced substantial noise, where, with minimal curation steps, 64% of IC_50_ measurements of the same compound-target pair differ by more than 0.3 log units, and 27% differ by more than one log unit.^8^ While their maximal curation approach requiring exact matching across ten assay metadata fields substantially reduces noise, such strict matching limits for data aggregation would result in sparse tasks with little overlap for multitask modeling.

CAPRICHO implements a complementary approach that prioritizes flexibility. To demonstrate this, we processed ChEMBL 32 using the targets investigated for the K_i_ data set from the Landrum and Riniker study^8^ with CAPRICHO’s quality control flags enabled. We aggregated the data for the 26 targets, considering compounds’ stereochemistries, flagged entries with undefined tetrahedral stereocenters, and applied some of the max-curation filters.^8^ Among these, --min-assay-overlap mirrors the criterion used by Landrum and Riniker^8^ to require a minimum number of compounds in common between compared assays. We applied it here to facilitate comparison with their study; the threshold controls the number of paired readouts available for estimating cross-assay agreement and should not be interpreted as a measure of assay or measurement quality. Those include registered document dates, assay size constraints (minimum 20, maximum 100 compounds per assay), mutation detection through mutation-related keywords in the assay description, and a required confidence score 9. To improve cross-assay comparability and prevent inappropriate aggregation, compound-target activities were grouped by assay type and cell type using the option --id-columns assay_type,assay_cell_type. Complete commands and parameter settings are provided in Supporting Text S1.

CAPRICHO’s K_i_ retrieval returned 97,981 measurements, which were aggregated into 48,184 data points; 2417 data points (5.0%) were measured in more than one distinct assay and yielded 11,679 pairwise comparisons before quality-flag removal. After excluding ChEMBL data-validity comments, potential duplicates, and identical-value pairs to define the naive baseline, 3611 comparisons remained (Spearman ρ = 0.70; 24.8% outside ± 1 log unit; Figure 1a). Figure 1b shows the effect of quality (flags 1–8; data_quality_comment) and processing (flags 9–10; data_processing_comment) steps on cross-assay comparability. Query construction rules are detailed in Supporting Text S2.

**Figure 1:**
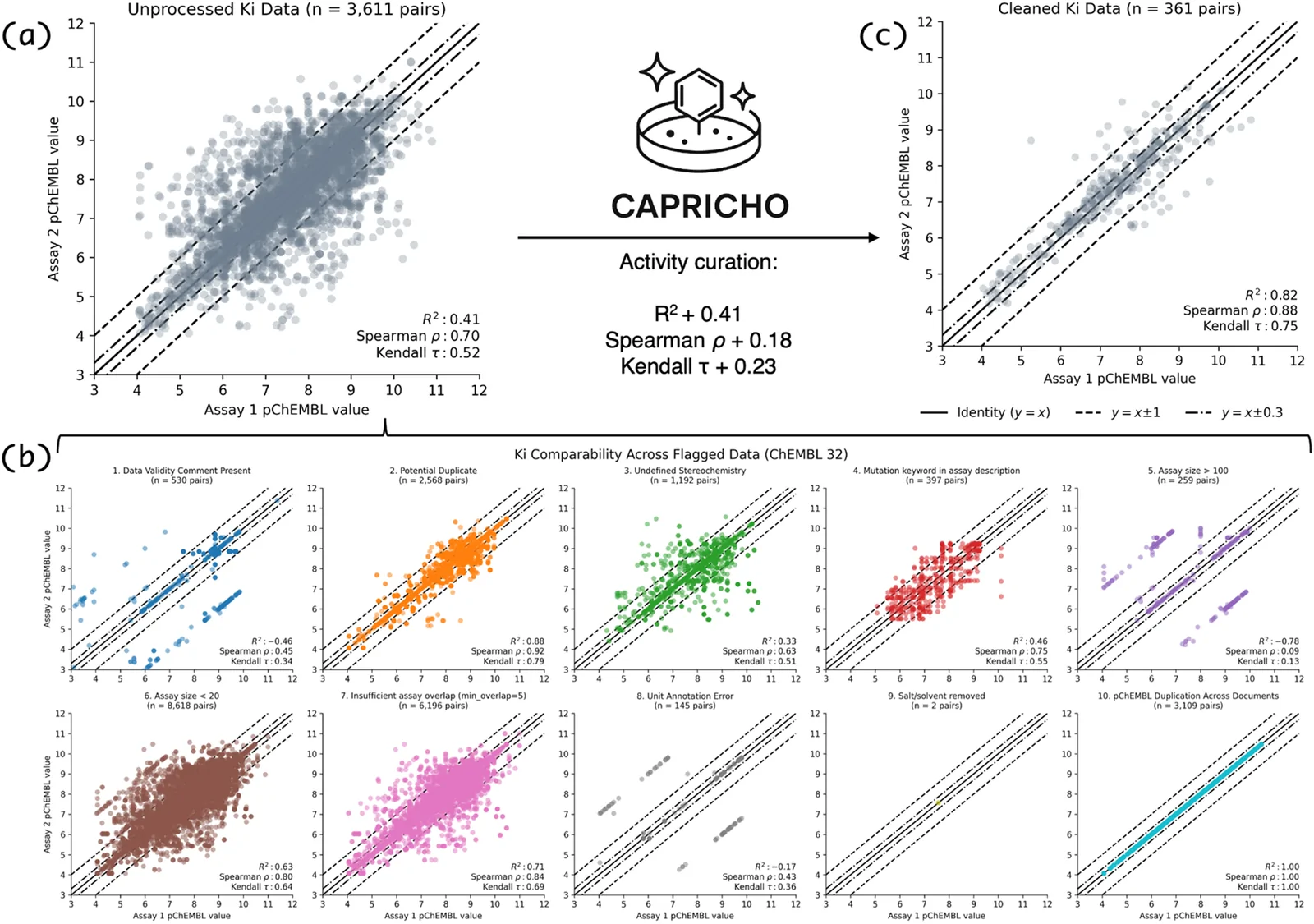
K_i_ data curation with CAPRICHO. (a) Aggregated K_i_ data with --id-columns assay_type,assay_cell_type and compound equality (connectivity), after excluding data validity comments, potential duplicates, and identical-value pairs, as a naive workflow. (b) Effect of individual quality flags (panels 1–8) and processing comments (panels 9–10) on cross-assay comparability: 1. Data validity comment present (ChEMBL-annotated quality issues); 2. Potential duplicate (ChEMBL-annotated); 3. Undefined stereochemistry; 4. Mutation keyword in assay description (nonwild-type targets); 5. Assay size >100 (likely high-throughput screens or nonliterature depositions); 6. Assay size <20 (specialized low-throughput conditions); 7. Insufficient assay overlap (<5 shared compounds, criterion from Landrum and Riniker^8^); 8. Unit annotation error (pChEMBL differing by exactly 3.0 or 6.0 log units across assays, indicating nM/μM mix-ups); 9. Salt/solvent removed; 10. pChEMBL duplication across documents (shown only for pairs where both assays share the annotation and report identical values). (c) Cleaned K_i_ data after removing entries flagged for data validity, potential duplicates, assay size violations, and cross-document duplication.

Examining the per-flag panels in Figure 1b reveals that quality flags differ dramatically in their impact on K_i_ cross-assay comparability. The most severe degradation is observed for data validity comments (panel 1; ρ = 0.45, 37.2% of pairs outside ± 1.0 log units) and large assays exceeding 100 compounds (panel 5; ρ = 0.09, 52.5% outside ± 1.0), both showing near-complete loss of agreement. The former confirms that ChEMBL’s own quality annotations identify genuinely problematic measurements, while the latter suggests that for the K_i_ values measured on those targets, high-throughput screening protocols may be poorly comparable with the smaller, focused medicinal chemistry assays. Notably, this pattern does not hold for IC_50_ data, where large assays retain good agreement (Figure S1, panel 5; ρ = 0.82), indicating that the impact of assay size on cross-assay comparability is readout-dependent. Unit annotation errors (panel 8; ρ = 0.43, 44.1% outside ± 1.0) also introduce substantial noise, producing extreme outliers where pChEMBL values differ by an exact multiple of 3.0 log units (3.0, 6.0, 9.0, ...). Conversely, small assays below 20 compounds (panel 6; ρ = 0.80, 15.9% outside ± 1.0 for K_i_) show substantially better summary metrics than data validity or large-assay flags, though the scatter plots reveal considerable spread that the correlation coefficients alone do not fully capture. For IC_50_, particularly, small-assay agreement is notably lower (Figure S1, panel 6; ρ = 0.60, 16.9% outside ± 1.0). This illustrates the value of pairing scatter plots with summary statistics: the visible spread, not captured by ρ alone, motivated removing small-assay data in the cleaned data set (Figure 1c), data-informed decision only possible when quality issues are preserved rather than silently filtered.

Other flags were less uniformly detrimental: undefined stereochemistry (*n* = 1192; ρ = 0.63; 18.0% outside ± 1.0) and mutation keywords (*n* = 397; ρ = 0.75; 26.7% outside ± 1.0) could be retained when coverage is prioritized, provided that the variability is accounted for. Cross-document duplicates showed perfect agreement (*n* = 3109; ρ = 1.00) but can inflate apparent confidence, whereas salt/solvent removal was rare (*n* = 2). These contrasts support end point- and model-specific decisions rather than uniform deletion.

By removing entries flagged for data validity issues, potential duplicates, assay size violations (both above and below threshold), and cross-document pChEMBL duplication, we generate a cleaned K_i_ activity subset with substantially improved comparability: Spearman ρ = 0.88, Kendall τ = 0.75, *R* ^2^ = 0.82 from 361 pairwise comparisons, with only 9.1% of remaining pairs outside the 1 log-unit threshold (Figure 1c). These metrics are consistent with the correlation levels achieved by the maximal curation approach of Landrum and Riniker (τ = 0.67 for their pruned K_i_ data set), demonstrating that CAPRICHO’s flag-based approach achieves comparable data quality while maintaining full transparency about curation decisions.

For IC_50_ data processed with equivalent parameters (39 targets), 142,440 retrieved measurements yielded 78,026 aggregated data points, of which 1603 (2.1%) were measured in more than one distinct assay. We observed substantially lower cross-assay agreement with Spearman ρ = 0.78, Kendall τ = 0.60, and *R* ^2^ = 0.50 across 552 pairwise comparisons, of which 16.7% fell outside the ± 1 log-unit range (Figure S1). This difference is consistent with the well-documented higher variability of IC_50_ measurements due to substrate concentration dependencies and other assay-specific factors. The pattern is consistent with both the original findings of Landrum and Riniker^8^ and CAPRICHO’s ability to expose data quality patterns that inform curation strategy selection.

Additionally, potential unit-annotation-error flags can support source verification rather than automatic deletion. CAPRICHO retains the ChEMBL activity, molecule, assay, target, and document identifiers associated with each candidate, while the ChEMBL document metadata provides the DOI needed to retrieve the original publication and Supporting Information. For molecule CHEMBL195218 against VEGFR-2 (CHEMBL279), activities 1512257 and 1512264, from assays CHEMBL829589 and CHEMBL829507, both map to document CHEMBL1144449, DOI 10.1021/jm0501275,^15^ source compound key 24, and source record 391924. The source table reports a VEGFR-2 IC_50_ of 62 nM for the corresponding (CH_2_)_3_SO_2_CH_3_ analogue, whereas the two ChEMBL activities report 62 and 0.062 nM.

Beyond data quality, CAPRICHO’s grouping strategy addresses a practical limitation of strict metadata matching for QSAR data set construction. Previously described maximal curation method used by Landrum and Riniker^8^ combines ten assay metadata fields, creating a separate data set partition for each unique combination. While valuable for characterizing cross-assay noise, this granularity fragments data sets into small partitions, likely containing too few compounds for reliable QSAR training. By grouping on assay_type and assay_cell_type, CAPRICHO produces coarser partitions that retain higher compound counts for modeling without compromising cross-assay comparability, as demonstrated above.

### Case Study 2: CYP Inhibition Multitask Data Set

To demonstrate CAPRICHO’s capabilities for multitarget ADME data preparation, we assembled a cytochrome P450 (CYP) inhibition data set spanning 14 CYP isoforms critical for drug metabolism. This data set was created based on the number of bioactivity data available for all cytochrome targets in ChEMBL, investigated with capricho explore commands (Supporting Text S3). A threshold of at least 500 data points with confidence scores of 8 or 9 was used for target selection. The following get command for data collection was then configured to maintain separation between activity types (IC_50_, K_i_, AC_50_, EC_50_, and Potency) during aggregation by including standard_type as an ID column. This separation leverages the data abundance of having multiple activity types available per target while preserving the higher cross-assay comparability inherent to K_i_ measurements, as demonstrated in Case Study 1. In a multitask modeling setting, each target-activity type combination becomes a separate task, allowing the model to learn across target affinities without mixing measurements from fundamentally different assay protocols. For the curation workflow, we used assay size constraints (minimum 5, maximum 100 compounds) and enabled stereochemistry-aware compound identification, flagging undefined stereocenters (Supporting Text S3). We lowered the minimum assay size to 5 (vs 20 in Case Study 1) to flag specialized CYP assays which may have less comparable readouts with higher-throughput screens while retaining a higher number of data points.

Processing 14 CYP targets from ChEMBL 36 yielded 75,678 aggregated compound-target-activity type combinations from 89,990 individual measurements using the local SQL backend. Of these, 8192 data points (10.8%) were measured in more than one distinct assay. Figure 2a shows cross-assay comparability for compounds measured in multiple assays after removing only data validity comments and potential duplicates, while Figure 2b shows the effect of additionally removing unit annotation errors, undefined stereochemistry, and assays below the size threshold. The cleaned set consisted of 2337 activity pairs and demonstrates improved agreement (*R* ^2^ = 0.55, Spearman ρ = 0.73, Kendall τ = 0.56; 11.6% outside ± 1 log unit; Figure 2b) compared to the 7988 activity pairs observed for unprocessed data (*R* ^2^ = 0.25, Spearman ρ = 0.62, Kendall τ = 0.46; 26.0% outside ± 1 log unit; Figure 2a).

**Figure 2:**
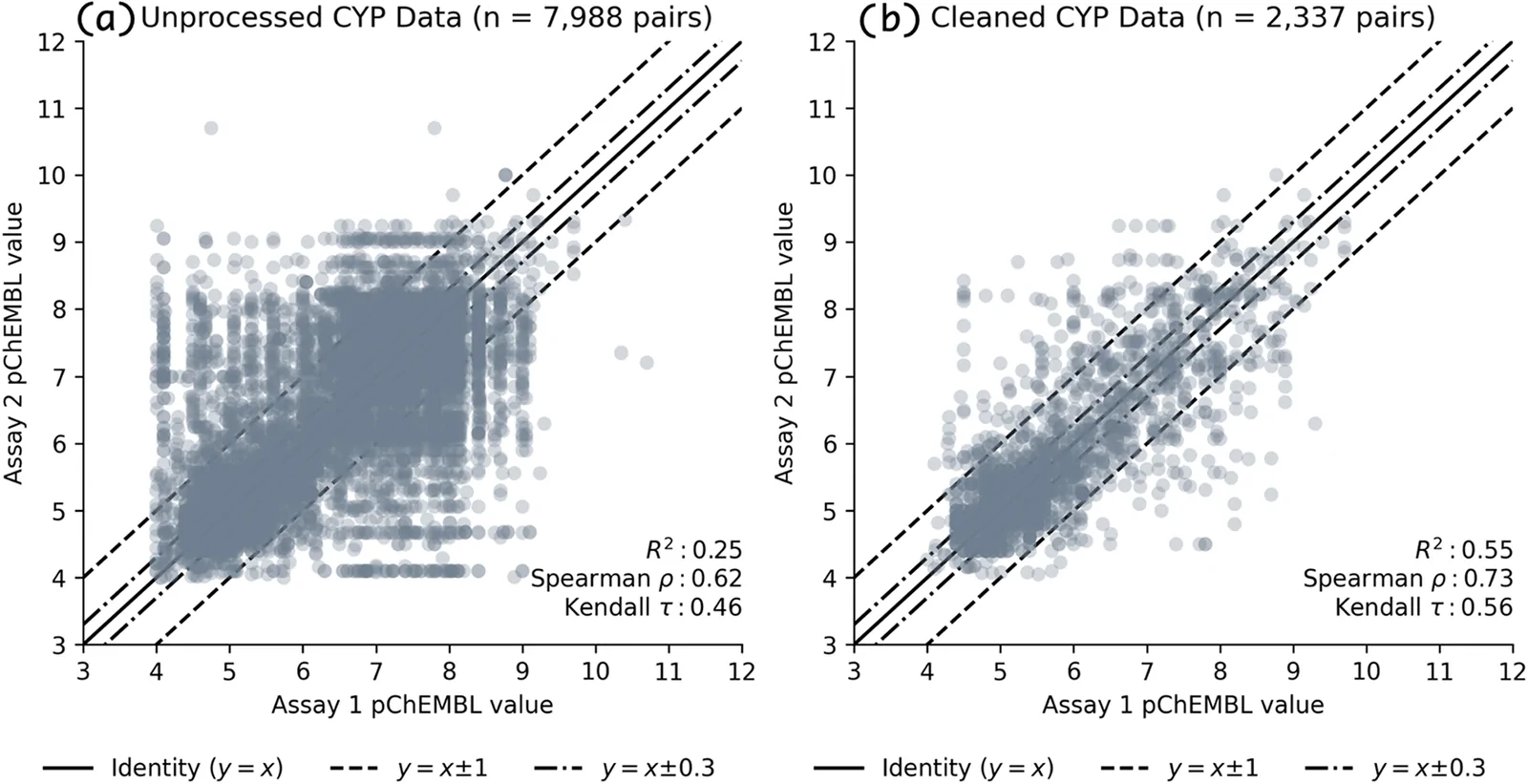
CYP multitask bioactivity curation with CAPRICHO. (a) Activity data after removing only data validity comments and potential duplicates, aggregated with --id-columns standard_type and compound equality (connectivity) for separation of IC_50_, K_i_, AC_50_, EC_50_, and Potency readouts. (b) Curated data after additionally removing entries flagged for unit annotation errors, undefined stereochemistry, and assays below size threshold.

In addition, CAPRICHO supports pivoting multitarget activities for multitask modeling with the prepare command, with compounds as SMILES (rows) and aggregated tasks as columns (target + defined identifier columns for aggregation). Figure S2 shows a Spearman correlation heatmap of the curated readouts across targets and bioactivity types. While cross-isoform correlations are generally positive, they are often not conserved across activity types, supporting the use of standard_type as an aggregation key.

### Case Study 3: Caco-2 Permeability with Unit Standardization

ADME property data in ChEMBL presents unique challenges beyond target affinity measurements, particularly unit heterogeneity and end point directionality. Caco-2 permeability measurements illustrate both problems: the same physical property (apparent permeability coefficient, Papp) is reported using 17 different unit representations across 296 assays, and critically, measurements can represent either the apical-to-basolateral (A to B) or the basolateral-to-apical (B to A) transport directions. Of these, nearly half (48.3%) of the 6477 measurements use semantically equivalent variants of 10^–6^ cm/s, while other permeability units (nm/s, cm/s, 10^–7^ cm/s) require conversion. However, unit standardization alone is insufficient: comparing A to B and B to A measurements is biologically wrong, as these represent fundamentally different transport mechanisms. Of the 6477 measurements, 2065 (31.9%; from 83 assays) lacked a recognized A-to-B or B-to-A term and were therefore classified as direction-unassigned. These measurements were retained in the end point and unit overview but excluded from direction-specific retrieval and all cross-assay comparability analyses.

CAPRICHO’s explore command enables identification of assay directionality from descriptions through custom SQL queries searching for the A to B and B to A keywords. Following this exploration, separate data retrieval commands can be configured for each direction, with unit conversion enabled to standardize heterogeneous unit representations, and grouping by standard units and assay cell type to maintain appropriate separation during aggregation. Complete query examples and parameter settings are provided in Supporting Text S4.

This direction-aware approach dramatically improves cross-assay comparability. When A to B and B to A measurements were naively combined, cross-assay correlation was poor (Spearman ρ = 0.49, Figure S3), reflecting the biological irrelevance of comparing absorption with efflux. After separation by direction and removal of data validity comments and potential duplicates, both data sets showed reasonable cross-assay agreement: A to B data (1870 compounds from 90 assays, 172 pairwise comparisons Figure S4a) showed *R* ^2^ = 0.33, Spearman ρ = 0.69 and Kendall τ = 0.51, while B to A data (777 compounds from 49 assays, 173 comparisons; Figure S5a) achieved *R* ^2^ = 0.52, Spearman ρ = 0.68, and Kendall τ = 0.50. Further curation by removing entries flagged for undefined stereochemistry improved the A to B agreement to *R* ^2^ = 0.48, Spearman ρ = 0.83 and Kendall τ = 0.64 (60 comparisons, Figure S4c) and B to A to *R* ^2^ = 0.79, Spearman ρ = 0.75 and Kendall τ = 0.57 (59 comparisons, Figure S5c). Figure 3 summarizes cross-assay comparability metrics before and after quality flag removal.

**Figure 3:**
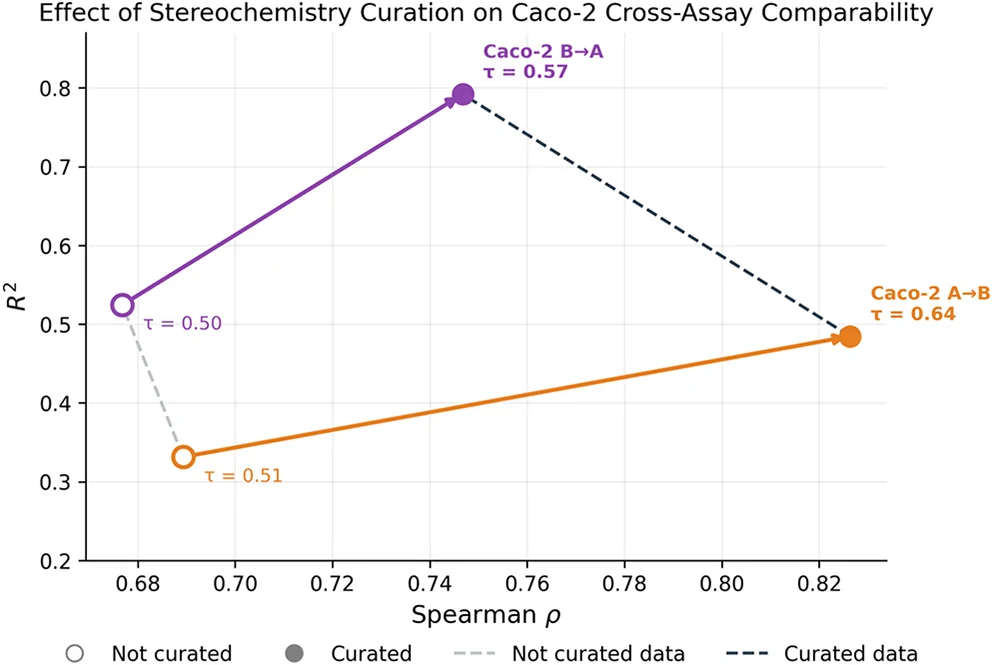
Effect of stereochemistry curation on Caco-2 cross-assay comparability. Each data set is represented by two points in Spearman ρ versus *R* ^2^ space: hollow markers indicate comparability before curation, and filled markers indicate comparability after removing entries with undefined tetrahedral stereocenters. Arrows show the trajectory of improvement for Caco-2 A→B (orange; 172 → 60 pairwise comparisons) and B→A (purple; 173 → 59 comparisons). Kendall τ values are annotated at each point. Dashed lines connect the before and after groups across data sets, illustrating that both transport directions benefit from stereochemistry curation, with B→A showing the largest *R* ^2^ improvement.

### Assessing Curation Effects Beyond Sample-Size Reduction

Because quality-flag removal substantially reduced the number of pairwise comparisons, we tested whether the observed improvements could be explained by sample-size reduction alone. One-sided matched-size randomization tests compared each cleaned set with 5,000 subsets sampled without replacement from its baseline at the cleaned sample size: CYP, *n* = 2337 of 7988; Caco-2 A→B, *n* = 60 of 172; and B→A, *n* = 59 of 173 (Figure S6). Empirical *p*-values were calculated as (*k* + 1)/5001, where *k* was the number of random subsets performing at least as well as the cleaned set. For CYP, targeted curation outperformed random removal for Spearman ρ, Kendall τ, *R* ^2^, and the fraction outside ± 1 log unit (*p* = 0.0002 for each), remaining significant after matching target–readout composition (all *p* ≤ 0.0008). For A→B, improvements in ρ and τ exceeded random removal (*p* = 0.0086 and 0.0132); *R* ^2^ was borderline under size-only matching (*p* = 0.0510) but improved after assay-pair matching (*p* = 0.0024), whereas the fraction outside ± 1 log unit did not improve (*p* = 0.954). For B→A, rank-correlation gains were not resolved (ρ: *p* = 0.111; τ: *p* = 0.103), although *R* ^2^ and mean unsigned error improved (*p* = 0.0020 and 0.0150). Thus, random data removal did not explain the CYP results or improved A→B rank agreement, whereas evidence for B→A remained metric-dependent.

## Conclusion

CAPRICHO consolidates flexible flagging, reproducibility, and metadata-aware aggregation into a single command-line tool for ChEMBL bioactivity curation. By preserving flagged data alongside processed values and representing cross-assay comparability both numerically and visually, CAPRICHO reveals that quality flags can have end point-specific effects and that no single statistic fully characterizes measurement agreement. Spearman ρ and Kendall τ describe rank consistency, whereas *R* ^2^ assesses agreement with the identity line and the fraction outside ± 1 log unit identifies large discrepancies. A high rank correlation can therefore coexist with poor absolute agreement, while summary metrics can conceal systematic offsets, heterogeneous spread, or influential clusters that remain apparent in the scatter plots. Considering these complementary views enables practitioners to make curation decisions grounded in the characteristics of their data, rather than applying uniform filtering rules across readout types.

The three case studies illustrate CAPRICHO’s practical utility across different data types and curation challenges. In Case Study 1, CAPRICHO’s flag-based approach achieved strong cross-assay agreement for both K_i_ (ρ = 0.88, τ = 0.75) and IC_50_ (ρ = 0.78, τ = 0.60) while using coarser assay and cell-type grouping than previously published fine-grained aggregation methods.^8^ In Case Study 2, CAPRICHO supported construction of a multitarget CYP data set while maintaining separation among activity types; the nested cleaned comparison reached ρ = 0.73 and τ = 0.56, with matched-size analysis showing improvements beyond those produced by random data removal. In Case Study 3, separating Caco-2 measurements by transport direction increased ρ from 0.49 for mixed directions to 0.69 for A→B and 0.68 for B→A before additional quality filtering, and subsequently to 0.83 and 0.75, respectively. The matched-size results nevertheless showed that the strength of evidence differed by direction and metric, reinforcing the need to evaluate comparability using multiple complementary measures.

Key features that distinguish CAPRICHO from existing workflows include: (1) transparent flagging with preservation of source measurements, enabling users to assess how each curation decision affects cross-assay comparability; (2) flexible grouping via --id-columns that balances data availability with aggregation granularity for both traditional and assay-aware modeling paradigms;^12^ and (3) automatic recipe generation for reproducibility. Rather than prescribing a universally appropriate set of filters, CAPRICHO allows users to inspect how each decision affects agreement, data set size, and coverage, thereby selecting a curation strategy appropriate to the end point and intended application. In this way, CAPRICHO makes bioactivity curation an explicit, inspectable, and reproducible part of data set construction rather than an opaque preprocessing step.

## Supplementary Material



## Data Availability

CAPRICHO is freely available under the MIT license at https://github.com/CDDLeiden/Capricho and on PyPI (pip install capricho). Case Study 1 used ChEMBL release 32; Cases 2 and 3 used ChEMBL release 36. Jupyter notebooks reproducing all analyses and figures presented in this manuscript are available at https://github.com/CDDLeiden/Capricho/tree/master/notebooks (case-1-*.ipynb through case-3-*.ipynb).

## Notes

CAPRICHO commands and parameter settings for retrieving and aggregating the K_i_ and IC_50_ data sets (Supporting Text S1); pair-construction and filtering rules used for Figure 1 (Supporting Text S2); CYP target-selection query and commands for constructing the CYP inhibition multitask data set (Supporting Text S3); Caco-2 transport-direction classification queries, assay lists, unit-conversion settings, and retrieval commands (Supporting Text S4); salt and solvent removal definitions along with the regular expression used in CAPRICHO (Supporting Text S5); IC_50_ curation and the effects of individual quality and processing flags on cross-assay comparability (Figure S1); Spearman correlation matrix of curated CYP bioactivity tasks (Figure S2); cross-assay comparability of Caco-2 permeability data with transport directions combined (Figure S3); Caco-2 apical-to-basolateral curation and comparability before and after stereochemistry filtering (Figure S4); Caco-2 basolateral-to-apical curation and comparability before and after stereochemistry filtering (Figure S5); and matched-size random-retention analyses of CYP and Caco-2 cross-assay comparability (Figure S6) (PDF)

## Abbreviations

QSAR: quantitative structure–activity relationship
CAPRICHO: ChEMBL Aggregation Package with Robust Inspection and Curation Handling Options
CYP: cytochrome P450
ADME: absorption, distribution, metabolism, and excretion
Papp: apparent permeability coefficient
SMILES: simplified molecular input line entry system
InChIKey: International Chemical Identifier Key
ECFP4: extended-connectivity fingerprints (diameter 4)
SQL: structured query language
API: application programming interface
pChEMBL: negative log_10_ of the molar activity value.
