# Supplementary material for: CAPRICHO: Interpretable Quality Flagging and Flexible ChEMBL Bioactivity Curation for QSAR Modeling
Source: J Chem Inf Model. 2026 Sep 23;66(19):12216–23. doi: 10.1021/acs.jcim.6c00802 (PMC13663851; doi:10.1021/acs.jcim.6c00802)
Supplement: Supplementary file 1 [file ci6c00802_si_001.pdf]

Supporting Information

CAPRICHO: Interpretable Quality Flagging and Flexible ChEMBL Bioactivity Curation for QSAR Modeling

David Alencar Araripe[1,2]; Srijit Seal[3,4,5,6,7], Olivier Béquignon[1], Gerard J.P. van Westen[1]*

[1] Division of Medicinal Chemistry, Leiden Academic Centre for Drug Research, Leiden University, Einsteinweg 55, 2333 CC Leiden, The Netherlands

[2] Department of Human Genetics, Leiden University Medical Center, Einthovenweg 20, 2333 ZC Leiden, The Netherlands

[3] Human Chemical Company, San Francisco, CA 94129, USA

[4] Department of Pharmaceutical Biosciences and Science for Life Laboratory, Uppsala University, Uppsala 75237, Sweden

[5] Broad Institute of MIT and Harvard, Cambridge, MA 02142, USA

[6] Centre for Molecular Informatics, Yusuf Hamied Department of Chemistry, University of Cambridge, Cambridge, CB2 1EW, UK

[7] STAR-UBB Institute, Babeş-Bolyai University, Cluj-Napoca 400084, Romania

*correspondence: gerard@gjpvanwesten.nl

Supplementary Text **3**
Supplementary Text S1 3
Supplementary Text S2 4
Supplementary Text S3 5
Supplementary Text S4 6
Supplementary Text S5 9
Supplementary Figures **10**

Supplementary Text

All commands were executed using CAPRICHO version 1.0.3 with ChEMBL release 36 via the local SQL backend (`chembl_downloader`). Case Study 1 used ChEMBL release 32 for comparability with the original study; Cases 2 and 3 used ChEMBL release 36. Recipe JSON files containing complete parameter specifications are automatically generated by CAPRICHO and all the commands are available in the GitHub repository.

Supplementary Text S1. Max-Curated K_i and IC_{50} Datasets: commands for data retrieval and aggregation

Ki dataset (26 targets):

```
capricho get \
  --target-ids CHEMBL205,CHEMBL214,CHEMBL217,CHEMBL218,CHEMBL224,CHEMBL226,\
CHEMBL228,CHEMBL233,CHEMBL234,CHEMBL237,CHEMBL243,CHEMBL244,CHEMBL251,\
CHEMBL253,CHEMBL255,CHEMBL256,CHEMBL259,CHEMBL261,CHEMBL264,CHEMBL313,\
CHEMBL3155,CHEMBL3242,CHEMBL3371,CHEMBL344,CHEMBL3594,CHEMBL4153 \
  --chirality \
  --drop-unassigned-chiral \
  --require-doc-date \
  --curate-annotation-errors \
  --max-assay-size 100 \
  --min-assay-size 20 \
  --min-assay-overlap 5 \
  --strict-mutant-removal \
  --calculate-pchembl \
  --confidence-scores 9 \
  --id-columns assay_type,assay_cell_type \
```

```
  --bioactivity-type Ki \
  --output-path curated-Ki.csv
```

IC_{50} dataset (39 targets): The IC_{50} dataset was processed with equivalent parameters, replacing --bioactivity-type Ki with --bioactivity-type IC50. Target ChEMBL IDs were extracted from the IC_{50} dataset definitions published by Landrum and Riniker (https://github.com/rinikerlab/overlapping_assays, datasets/IC50_datasets.yaml). The complete target list and commands are available in the CAPRICHO repository https://github.com/CDDLeiden/Capricho.

Supplementary Text S2: Figure 1. Construction Rules

To construct each panel, pairwise comparisons are made for compounds measured in two or more assays and then filtered according to the nature of each flag. For quality flags tied to individual assay characteristics (e.g., undefined stereochemistry, assay size violations), pairs are shown where at least one assay carries the flag, so that flagged measurements are compared against the broader dataset rather than only against each other. For instance, comparing small assays exclusively to other small assays would not capture how their measurements relate to the rest of the data. For relational flags (e.g., potential duplicates, unit annotation errors, and cross-document pChEMBL duplication), both assays in the pair must carry the flag, since these issues are only meaningful to inspect when both sides share the annotation. For cross-document duplication, pairs are further restricted to those reporting identical pChEMBL values. Processing comments (panels 9 and 10) are shown in isolation from quality flags to separate the effect of data transformations

from data quality issues. In all quality flag panels except data validity, entries with data validity comments are excluded to avoid conflating their effect with the flag under inspection.

Supplementary Text S3: CYP Inhibition Multitask Dataset

CYP targets were selected using `capricho explore` to identify all cytochrome P450 targets in ChEMBL with at least 500 bioactivity measurements at confidence scores 8 or 9:

```
capricho explore --query "
SELECT td.chembl_id AS target_chembl_id,
       td.pref_name AS target_name,
       COUNT(*) AS n_activities
FROM activities act
JOIN assays a ON act.assay_id = a.assay_id
JOIN target_dictionary td ON a.tid = td.tid
WHERE td.pref_name LIKE '%Cytochrome P450%'
  AND a.confidence_score IN (8, 9)
GROUP BY td.chembl_id, td.pref_name
HAVING COUNT(*) >= 500
ORDER BY n_activities DESC
"
```

The ChEMBL IDs of the identified targets with the command above were then used for data retrieval and aggregation with the following command:

```
capricho get \
  --target-ids CHEMBL340,CHEMBL289,CHEMBL3397,CHEMBL3622,CHEMBL3356,\
CHEMBL2722,CHEMBL1908,CHEMBL3379,CHEMBL3978,CHEMBL4878,\
CHEMBL2231,CHEMBL3721,CHEMBL5282,CHEMBL4729 \
  --drop-unassigned-chiral \
  --chirality \
```

```
  --min-assay-size 5 \
  --max-assay-size 100 \
  --assay-types A,B,F \
  --id-columns standard_type \
  --output-path cyp_chembl_data.csv
```

Supplementary Text S4 Caco-2 Permeability

Caco-2 permeability assays were classified by transport direction using `capricho explore` queries over assay descriptions. Among 6,477 candidate measurements, 2,065 (31.9%) from 83 of 296 assays lacked a recognized A→B or B→A term. No direction was inferred: these direction-unassigned measurements remained in the endpoint and unit overview but were excluded from both direction-specific datasets and every comparability analysis. The full discovery script covers A→B, B→A, bidirectional, and direction-unassigned assays.

```
capricho explore --query "
SELECT a.chembl_id AS assay_chembl_id,
       a.description,
       COUNT(DISTINCT act.molregno) AS n_compounds
FROM assays a
JOIN activities act ON a.assay_id = act.assay_id
WHERE LOWER(a.description) LIKE '%caco%'
  AND act.standard_units IS NOT NULL
  AND (   LOWER(a.description) LIKE '%apical to basolateral%'
       OR LOWER(a.description) LIKE '%apical to basal %'
       OR LOWER(a.description) LIKE '%apical side to basolateral%'
       OR LOWER(a.description) LIKE '%(a to b)%'
       OR LOWER(a.description) LIKE '%a-to-b%'
```

```
  )
  AND NOT (   LOWER(a.description) LIKE '%basolateral to apical%'
           OR LOWER(a.description) LIKE '%basal to apical%'
           OR LOWER(a.description) LIKE '%b to a%'
  )
GROUP BY a.chembl_id, a.description
HAVING COUNT(DISTINCT act.molregno) >= 5
ORDER BY n_compounds DESC
"
```

The following command was used for fetching Caco-2 A→B (absorption) permeability data (92 assays):

```
capricho get \
  --assay-ids CHEMBL3529272,CHEMBL3430218,CHEMBL3529276,CHEMBL3788414,\
CHEMBL4618331,CHEMBL967838,CHEMBL1010166,CHEMBL2399272,CHEMBL5535226,\
CHEMBL3102303,CHEMBL3413801,CHEMBL5226219,CHEMBL5367468,CHEMBL1936906,\
CHEMBL4626019,CHEMBL2061608,CHEMBL4718821,CHEMBL4822451,CHEMBL2404820,\
CHEMBL3791483,CHEMBL1781718,CHEMBL2182708,CHEMBL3529287,CHEMBL1777560,\
CHEMBL1810827,CHEMBL1954393,CHEMBL3135115,CHEMBL3224753,CHEMBL3529284,\
CHEMBL4016544,CHEMBL898578,CHEMBL1780678,CHEMBL4353732,CHEMBL1953550,\
CHEMBL2026558,CHEMBL2186064,CHEMBL2321450,CHEMBL4775534,CHEMBL3776213,\
CHEMBL4144179,CHEMBL930649,CHEMBL1059634,CHEMBL3396820,CHEMBL4616467,\
CHEMBL4842267,CHEMBL5243021,CHEMBL1119778,CHEMBL1657599,CHEMBL2382568,\
CHEMBL3411223,CHEMBL4266470,CHEMBL4322186,CHEMBL1775251,CHEMBL2175485,\
CHEMBL3750318,CHEMBL3294034,CHEMBL3295547,CHEMBL4481174,CHEMBL4672670,\
CHEMBL967979,CHEMBL2424026,CHEMBL3124084,CHEMBL3620852,CHEMBL4143981,\
CHEMBL4157520,CHEMBL4414795,CHEMBL5615768,CHEMBL3368176,CHEMBL3371469,\
CHEMBL3829697,CHEMBL4351258,CHEMBL4672721,CHEMBL5112808,CHEMBL5166088,\
CHEMBL5340432,CHEMBL5349017,CHEMBL5384114,CHEMBL923278,CHEMBL935710,\
```

```
CHEMBL1780502,CHEMBL1780839,CHEMBL2399058,CHEMBL2400832,CHEMBL3300842,\
CHEMBL3779198,CHEMBL3860793,CHEMBL4731880,CHEMBL5217583,CHEMBL5340614,\
CHEMBL5638895,CHEMBL921232,CHEMBL923495 \
  --assay-types A \
  --confidence-scores 0,1,2,3,4,5,6,7,8,9 \
  --value-column standard_value \
  --id-columns standard_units,assay_cell_type \
  --convert-units \
  --drop-unassigned-chiral \
  --output-path caco2_a_to_b.csv
```

The following command was used for fetching Caco-2 B→A (efflux) permeability data (49 assays):

```
capricho get \
  --assay-ids CHEMBL3529273,CHEMBL3529277,CHEMBL3413802,CHEMBL1936907,\
CHEMBL2061609,CHEMBL4718841,CHEMBL3529288,CHEMBL3529285,CHEMBL2026704,\
CHEMBL2321447,CHEMBL3396835,CHEMBL1657600,CHEMBL4322187,CHEMBL3750319,\
CHEMBL3102302,CHEMBL3294035,CHEMBL4157521,CHEMBL5349018,CHEMBL5384115,\
CHEMBL923279,CHEMBL1780503,CHEMBL2400831,CHEMBL4731881,CHEMBL5388553,\
CHEMBL5638896,CHEMBL921231,CHEMBL923494,CHEMBL1100523,CHEMBL1959677,\
CHEMBL3368149,CHEMBL4477890,CHEMBL5141815,CHEMBL5503593,CHEMBL1060844,\
CHEMBL1943181,CHEMBL2026005,CHEMBL4037922,CHEMBL944381,CHEMBL1803338,\
CHEMBL2148941,CHEMBL2350101,CHEMBL3419538,CHEMBL4357336,CHEMBL4373230,\
CHEMBL4380529,CHEMBL5111948,CHEMBL5128826,CHEMBL5153793,CHEMBL967839 \
  --assay-types A \
  --confidence-scores 0,1,2,3,4,5,6,7,8,9 \
  --value-column standard_value \
  --id-columns standard_units,assay_cell_type \
```

```
  --convert-units \
  --drop-unassigned-chiral \
  --output-path caco2_b_to_a.csv
```

Supplementary Text S5: Salt and Solvent Removal

After `chembl_structure_pipeline` standardization, CAPRICHO splits each SMILES on "`.`" and removes only complete components exactly matching its documented ion and solvent list: sodium, potassium, lithium, chloride, bromide, fluoride, iodide, calcium, magnesium, zinc, hydroxide, water, ammonia, pyridine, N,N-dimethylformamide, benzoate, butyrate, pentanoate, perchlorate, tetraphenylborate, and nitric oxide. Original ChEMBL SMILES remain in `canonical_smiles` and removals are flagged in `data_processing_comment`.

```
MIXTURE_REGEX = re.compile(
    r"^("  # start of the string
    r"Na\+?|Cl\-?|Br\-?|K\+?|F\-?|I\-?|Ca\+{2}?|Mg\+{2}?|Zn\+{2}?|OH\-?|"
# Salts (bare)
    r"\[Na\+\]?|\[K\+\]?|\[Cl-\]?|\[Br-\]?|\[I-\]?|"  # Salts (bracketed,
monovalent)
    r"\[Zn\+2\]?|\[Ca\+2\]?|\[Mg\+2\]?|\[Li\+\]?|"  # Salts (bracketed,
divalent + Li)
    r"CCCC\(=O\)\[O-\]|"  # Butyrate
    r"CCCCC\(=O\)\[O-\]|"  # Pentanoate
    r"O=C\(\[O-\]\)c1ccccc1|"  # Benzoate (RDKit-canonical)
    r"\[O-\]\[Cl\+3\]\(\[O-\]\)\(\[O-\]\)\[O-\]|"  # Perchlorate [O-
][Cl+3]([O-])([O-])[O-]
    r"c1ccncc1|"  # Pyridine (RDKit-canonical)
    r"CN\(C\)C=O|"  # N,N-Dimethylformamide (RDKit-canonical)
    r"\[N\]=O|"  # Nitric oxide (RDKit-canonical)
    r"c1ccc\(\[B-\]\(c2ccccc2\)\(c2ccccc2\)c2ccccc2\)cc1|"  #
Tetraphenylborate
    r"^O$|^N$"  # Match full strings for O and N
    r")$"  # end of the string
)
```

Supplementary Figures

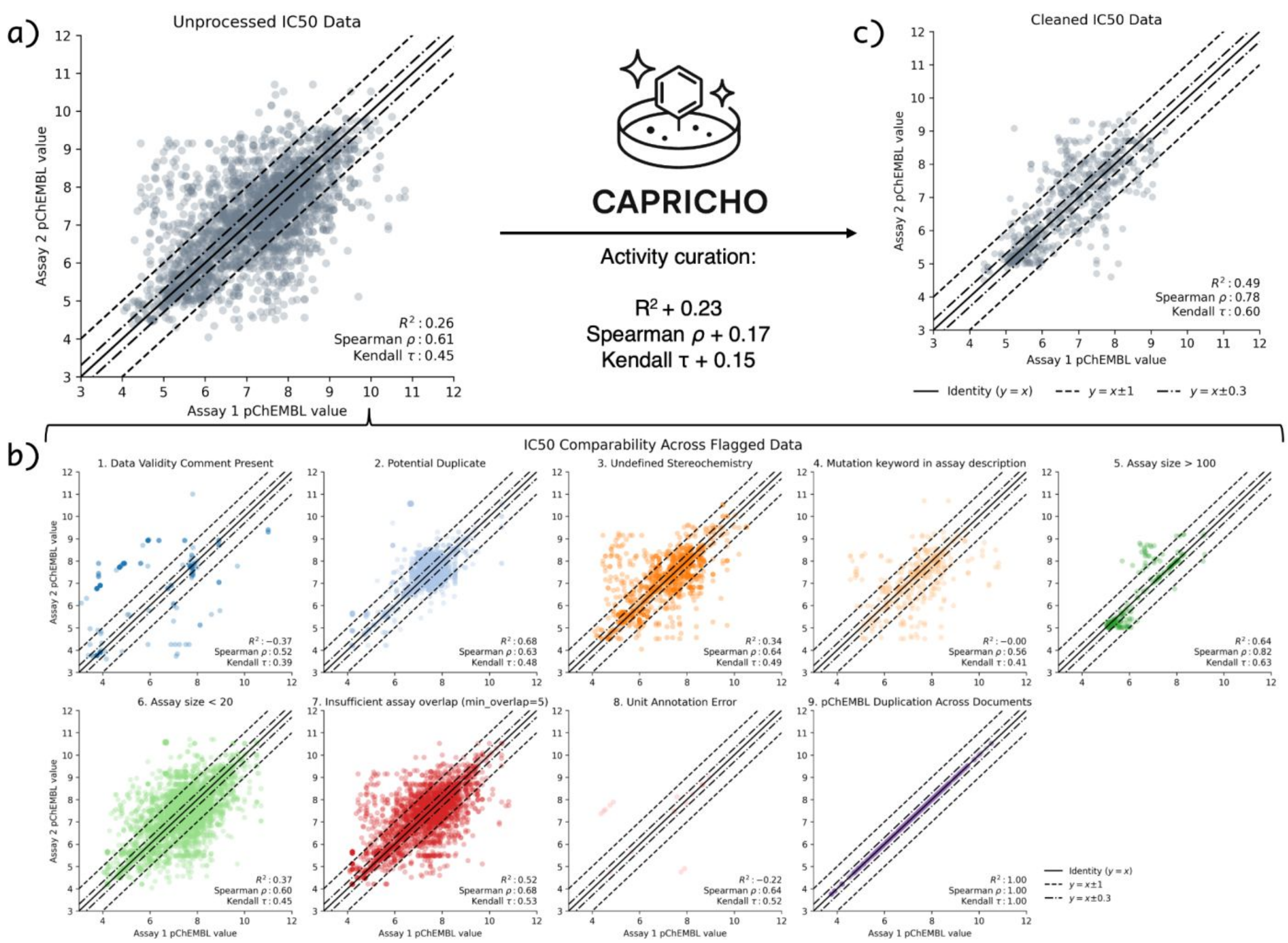

Figure S1. Activity curation process with CAPRICHO, showcasing the support for tracking different quality and processing comments. a) Fetched IC_{50} data prior to quality control filtering, aggregated with the conditions `--id-columns assay_type,assay_cell_type`, in addition to compound equality. Data-validity comments, potential duplicates, and identical-value pairs were excluded to exemplify a naive data aggregation workflow. b) Multi-panel view showing the effect of individual quality flags (panels 1–8) and processing comments (panel 9) on cross-assay comparability: 1. Data Validity Comment Present — entries with ChEMBL-annotated data quality issues; 2. Potential Duplicate — measurements flagged as potential duplicates by

ChEMBL; 3. Undefined Stereochemistry — compounds with unresolved stereocenters; 4. Mutation Keyword in Assay Description — assays containing mutation-related keywords suggesting non-wild-type targets; 5. Assay Size > 100 — assays exceeding 100 compounds, which may represent high-throughput screens or ChEMBL data depositions other than literature curation; 6. Assay Size < 20 — assays with fewer than 20 compounds, lower-throughput or specialized assay conditions designed to address specific research questions, thereby lowering comparability with broader screening assays; 7. Insufficient Assay Overlap — assays sharing fewer than 5 compounds with any other assay, a criterion introduced by Landrum and Riniker and supported here for demonstration purposes; 8. Unit Annotation Error — measurements where the same molecule shows pChEMBL values differing by an exact multiple of 3.0 log units across assays, indicating a unit annotation error (e.g., nM reported as µM or mM and vice versa); 9. pChEMBL Duplication Across Documents — identical compound-activity value pairs appearing in different publications, shown only for pairs where both assays share this annotation and report identical values. c) Cleaned IC_{50} activity values after removing entries flagged for data validity issues, potential duplicates, small assay size violations, assays containing mutation-related keywords, compounds with undefined stereochemistry and assays with detected activity annotation errors.

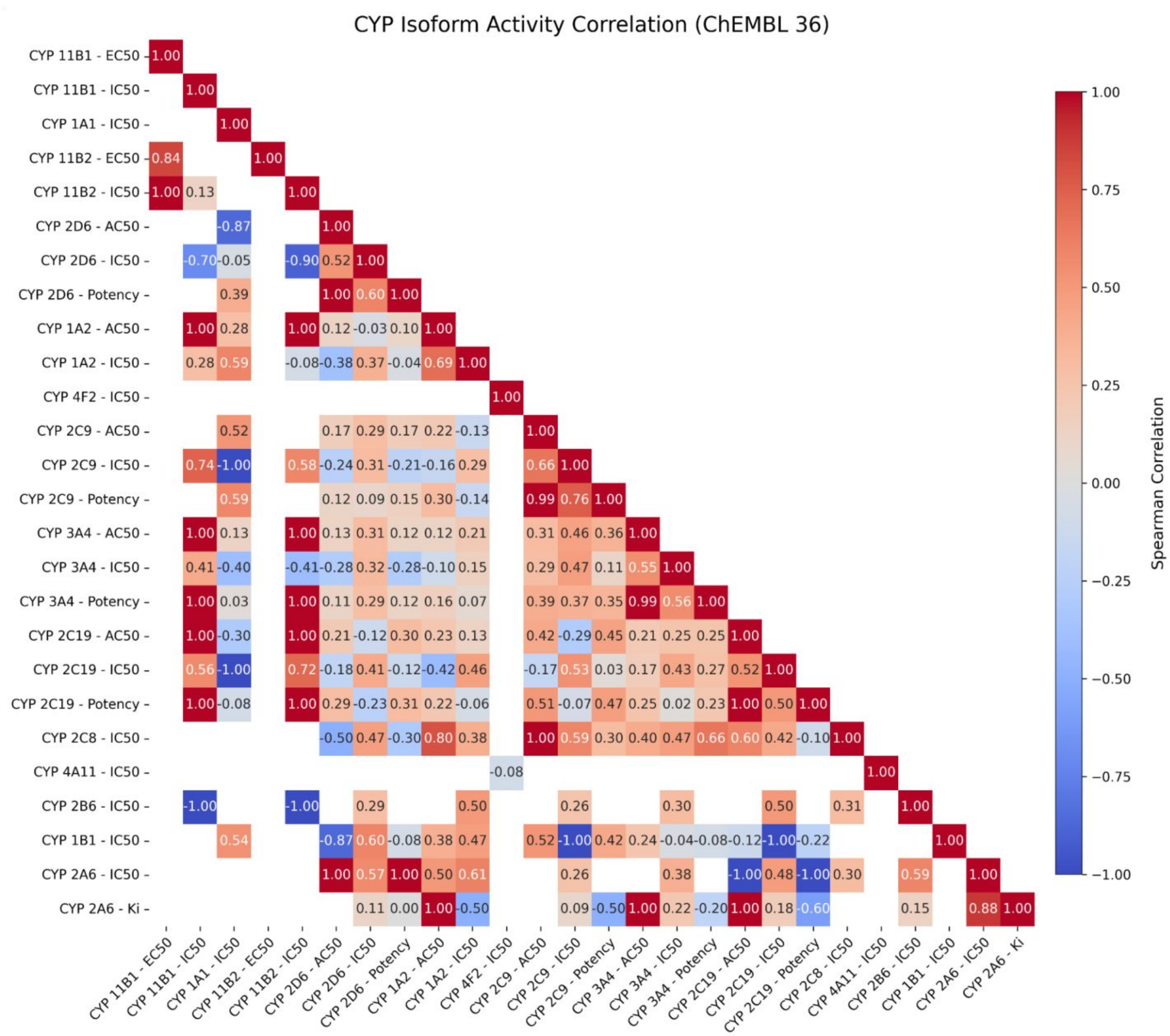

Figure S2. Spearman correlation matrix of the curated bioactivity tasks, showing expected relationships between CYP subfamily members: high correlations within the CYP2C subfamily (2C9, 2C19, 2C8) and between CYP1A isoforms, while CYP3A4 shows more variable correlations reflecting its broader substrate specificity. Only tasks with at least 100 curated compounds are shown.

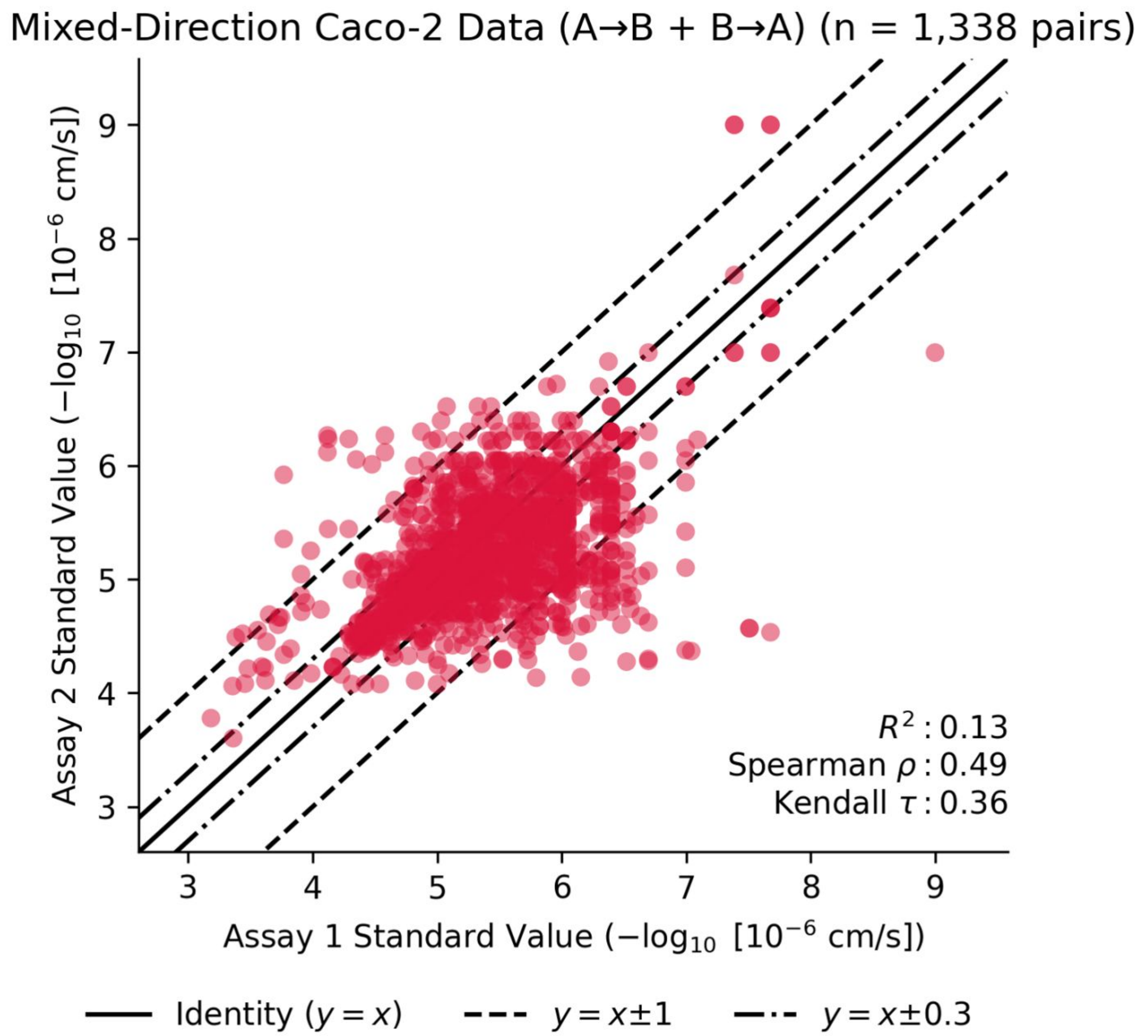

Figure S3. Cross-assay comparability of Caco-2 permeability data when A to B (apical to basolateral) and B to A (basolateral to apical) measurements are naively combined without direction separation. The poor agreement (Spearman ρ = 0.49) reflects the biological irrelevance of comparing absorption and efflux transport measurements, motivating the direction-aware curation approach described in Case Study 3.

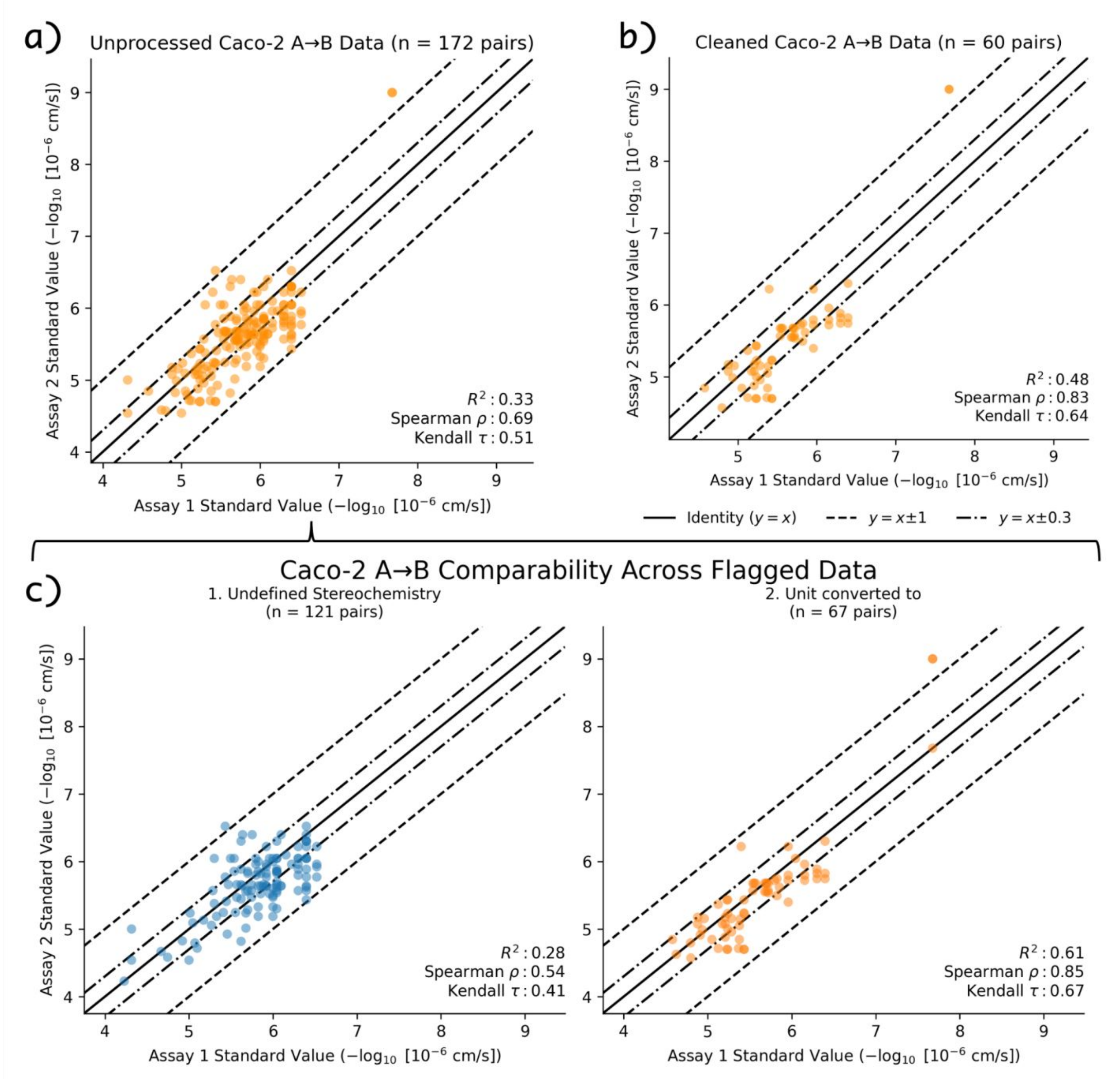

Figure S4. Activity curation of Caco-2 apical to basolateral permeability data with CAPRICHO. a) Fetched Caco-2 A to B data prior to quality control filtering. b) Multi-panel view showing the effect of individual quality flags (panel 1) and processing comments (panel 2) on cross-assay comparability: 1. Undefined Stereochemistry — compounds with unresolved stereocenters; 2. Unit conversion — activity values (`standard_value`) reported in a unit (`standard_units`)

compatible with $-\log_{10}$ [10^{-6} cm/s] conversion. c) Cleaned Caco-2 A to B activity values upon removal of compounds with undefined stereochemistry.

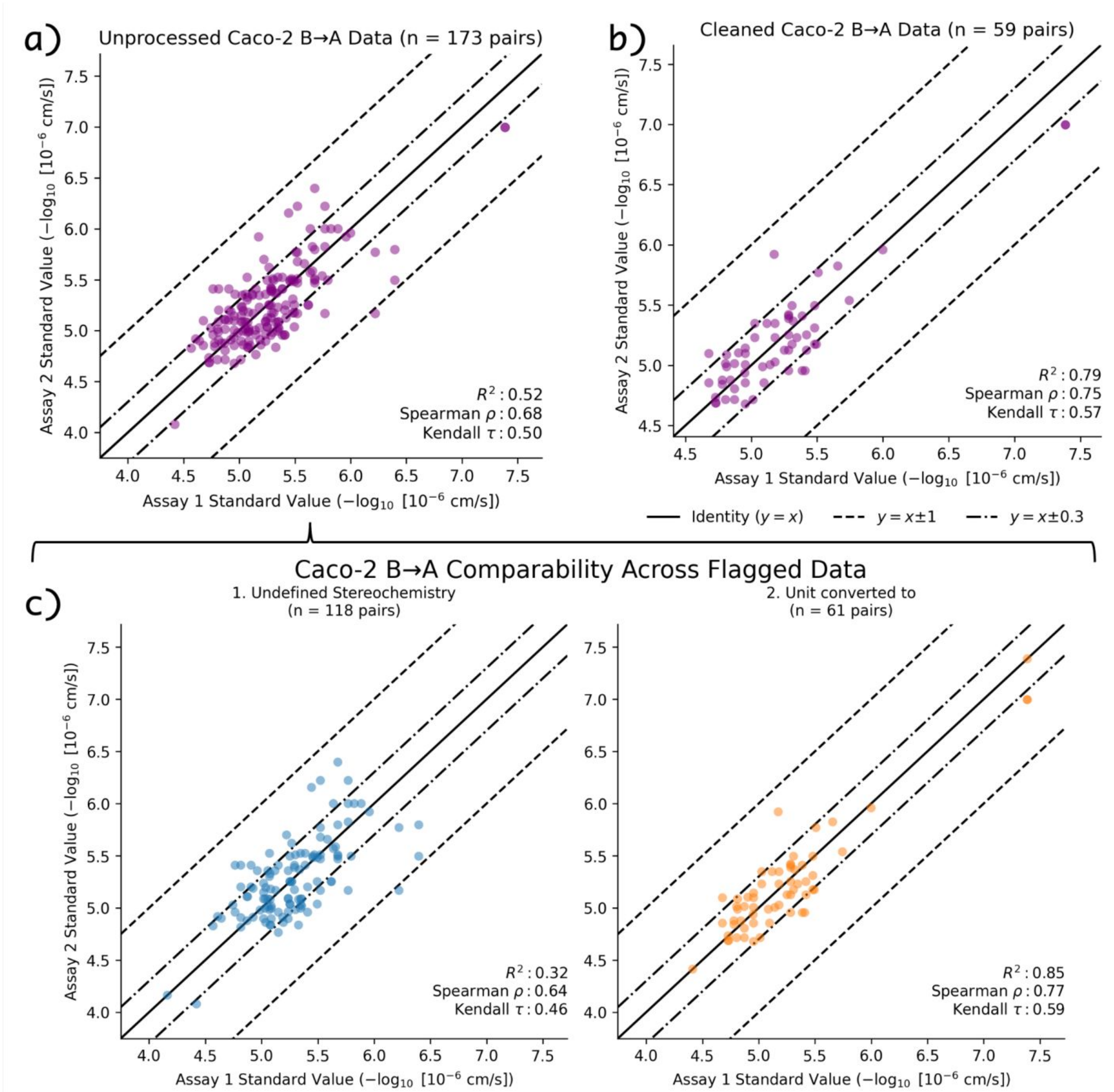

Figure S5. Activity curation of Caco-2 basolateral to apical permeability data with CAPRICHO. a) Fetched Caco-2 B to A data prior to quality control filtering. b) Multi-panel view showing the

effect of individual quality flags (panel 1) and processing comments (panel 2) on cross-assay comparability: 1. Undefined Stereochemistry — compounds with unresolved stereocenters; 2. Unit conversion — activity values (`standard_value`) reported in a unit (`standard_units`) `compatible with` $-\log_{10}$ [10^{-6} cm/s] conversion. c) Cleaned Caco-2 B to A activity values upon removal of compounds with undefined stereochemistry.

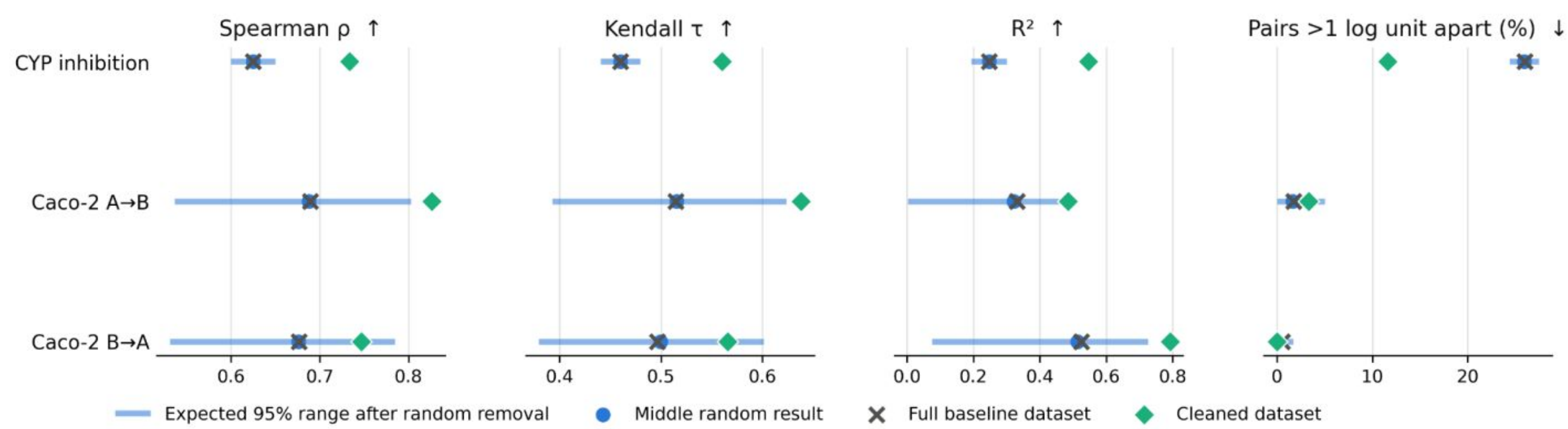

Figure S6. Matched-size random-retention analysis of CYP and Caco-2 cross-assay comparability. For each dataset, 5,000 subsets were sampled without replacement from the baseline at the cleaned sample size: CYP inhibition, 7,988 → 2,337 comparisons; Caco-2 A→B, 172 → 60; and Caco-2 B→A, 173 → 59. Each row represents one dataset. Gray crosses show the statistic for the complete baseline, blue circles show the median after random removal, and blue horizontal lines span the 2.5th–97.5th percentiles of the matched-size random-retention distribution. Green diamonds show the statistic for the actual cleaned dataset. Arrows indicate the favorable direction for each metric (i.e. up when a higher metric is better). A green diamond outside the blue interval in the favorable direction indicates that targeted removal produced a result beyond the central 95% of results obtained through random removal alone.

The figure shows the size-matched analysis; one-sided empirical *p*-values and the additional task- or assay-pair-matched analyses are reported in the main text.
